# Use of Traditional Korean Medicine by Patients with Musculoskeletal Disorders

**DOI:** 10.1371/journal.pone.0063209

**Published:** 2013-05-02

**Authors:** Bo-Ram Wang, In Young Choi, Kwang-Jum Kim, Young Dae Kwon

**Affiliations:** 1 Catholic Institute for Healthcare Management and Graduate School of Healthcare Management and Policy, the Catholic University of Korea, Seoul, Korea; 2 Department of Humanities and Social Medicine, College of Medicine and Catholic Institute for Healthcare Management, the Catholic University of Korea, Seoul, Korea; CUNY, United States of America

## Abstract

**Background:**

South Korea has maintained a dual healthcare delivery system that incorporates both traditional Korean and Western medicine. In this research, we identified the determinants of the frequency of using traditional Korean medicine among musculoskeletal patients, who are known to be the most frequent users of complementary and alternative medicine.

**Methods:**

In this research, we reviewed 2 consecutive years of nationally representative survey data from the 2008 and 2009 Korea Health Panel Survey. We analyzed the utilization of outpatient services by musculoskeletal patients within 12 months of the 2009 survey date. A two-part model was used because some patients did not use traditional Korean medicine and skewness was present in the data on traditional Korean medicine use. In the first part, logistic regression analysis was performed to investigate the use of traditional Korean medicine. In the second part, multiple regression analysis was performed to analyze the frequency of traditional Korean medicine usage among the subjects who visited traditional Korean medical institutions.

**Results:**

The frequency of traditional Korean medicine usage was positively associated with ages of 40–49 years and over 60, restrictions on daily life, a greater number of chronic diseases, not being hospitalized, and more frequent visits (more than five times) to conventional hospitals or clinics for musculoskeletal disorders.

**Conclusions:**

The important determinants of the frequency of traditional Korean medicine usage were age, activity restrictions, the number of chronic diseases, hospitalization history, and the number of visits to conventional hospitals for musculoskeletal disorders. The results contribute to our understanding of the characteristics of traditional Korean medicine users and may be used as a basic resource for related policymaking by government officials and medical professionals.

## Introduction

Interest in complementary and alternative medicine (CAM) has been increasing worldwide. In the United States, Congress passed legislation that established the Office of Alternative Medicine (OAM) within the National Institutes of Health in 1992. Congress later elevated the status of the OAM by establishing the National Center for Complementary and Alternative Medicine (NCCAM) in 1998 to facilitate the study and evaluation of CAM practices and to disseminate the resulting information to the public [1]. According to previous research, approximately 1/3 of U.S. adults have used CAM [2], [3]. The use of CAM has also been emerging in European countries since the 1980s [4], [5]. According to a CAM utilization survey in 2004 among cancer patients in 14 European countries, more than 40% of the patients in the Czech Republic, Switzerland, and Belgium had used CAM [6].

Unlike the Western countries where interest in CAM has been rapidly increasing in recent years, South Korea has maintained a dual healthcare delivery system that incorporates both traditional Korean and Western medicine. Traditional Korean medicine is recognized as a legitimate healthcare delivery system in Korea, but it is considered a form of CAM from the perspective of conventional Western medicine [7], [8]. Traditional Korean medicine has been an integral part of the prevailing practice and belief systems throughout Korea’s history. Western medicine, on the other hand, was introduced to Korea by missionaries from the U.S. and Western-trained doctors from Japan at the end of the 19^th^ century. In the past, the Korean government maintained policies that strengthened Western medicine while ignoring and suppressing traditional Korean medicine [9]. However, an official dual healthcare delivery system has been maintained to date by having separate physician’s licenses, physician’s educational institutions, and medical facilities for Western and traditional Korean medicine. Despite this dual system, Western medicine has a numerical advantage in Korea. At the end of 2010, there were 101,371 Western physicians and 19,132 traditional Korean physicians, and there were 29,946 conventional hospitals and clinics and 12,212 traditional Korean medical institutions in Korea [10]. Korea’s National Health Insurance (NHI) coverage for traditional medicine compared to conventional medicine is very low because it only covers check-ups, inpatient care, a small part of traditional tests and diagnosis, a small part of herbal medications, and a few kinds of traditional therapeutic procedures such as acupuncture and cupping [11]. Although only a small proportion of care provided by traditional Korean physicians or medical institutions is covered by the NHI system, the annual percentage of traditional Korean medicine utilization has remained steady at approximately 5%, according to NHI claims [11].

The changing disease patterns and the increase in the need for chronic disease management have caused the increasing interest in and use of CAM across the globe. As disease patterns have shifted from acute infections to chronic and degenerative diseases, medical services have changed from short-term treatment to ongoing management. Conventional medicine, however, is limited in its ability to manage chronic and degenerative diseases and treat various intractable diseases. During long-term treatment or management, patients suffer from drug side effects, dissatisfaction with therapeutic effects and rapid increases in cost [12]. To overcome these limitations of Western medicine, CAM has become increasingly accepted in disease treatment and the promotion of health and wellness [13].

Various researches have been conducted on the characteristics of CAM users and the determinants of CAM use. Some researches show that CAM utilization is influenced by an individual’s personality, family and friends, and socioeconomic factors, such as race/culture, education, and economic level [13]–[19]. However, these researchers analyzed utilization of CAM by including various treatments or approaches in one CAM category [13], [15], [20]. In addition, the same factors affected CAM utilization differently depending on the target region or country [21]–[23]. The number of researches addressing the determinants of CAM utilization with respect to frequency or cost is very low compared to the number of researches on the factors influencing CAM use or selection. Therefore, additional research is required to more accurately determine the factors that influence CAM utilization. In general, it is known that the severity or type of disease significantly affects the choice of health care provider or service [24], [25]. Previous research shows that CAM utilization is most strongly associated with musculoskeletal disorders [17], [18], [26], [27]. Thus, by controlling for the disease or the purpose of CAM utilization, it should be possible to determine the specific individual characteristics that affect CAM use.

In this research, we analyzed the determinants of the frequency of traditional Korean medicine usage by musculoskeletal patients, who are known to be the most frequent users of CAM. Nationally representative data from the Korea Health Panel Survey (KHPS) were used for the analysis.

## Methods

### 1. Data

The Korea Institute for Health and Social Affairs and the National Health Insurance Corporation (NHIC) have been conducting the KHPS since 2008 to produce representative and reliable statistics about the use of health services and health expenditures on a national scale. The survey sample was selected using 2-stage cluster stratified sampling with probability proportionate to size (i.e., by drawing a sample survey unit in stage 1 and then drawing sample households within the sample survey unit in stage 2). The KHPS is a longitudinal study with an initial survey sample of 9,105 households in 2008. During the follow-up surveys, some sample households were eliminated naturally and the total number of survey samples in 2009 was 7,500 [28]. The survey includes various items related to health service use patterns and health expenditures, so the study is able to analyze the use of Western and traditional Korean medical services and the frequency of use by the target households. In this study, we integrated the 2008 (1^st^ and 2^nd^ rounds) and 2009 KHPS data on the use of outpatient services and we analyzed the data of the selected samples who had participated in every survey in 2008 and 2009.This study was approved by the Institutional Review Board (IRB) of the Catholic University of Korea with a waiver for informed consent. There are no identified risks to the subjects of this study because the survey data were analyzed anonymously.

### 2. Subjects

The subjects in this study were musculoskeletal patients who had used outpatient services within 12 months of the 2009 survey date. We analyzed their use of traditional Korean medicine and the frequency of their usage. We selected the subjects from the KHPS data by first selecting people who had used outpatient services and then limiting the utilization period to within 12 months of the survey date in 2009. Users under 20 years of age were excluded because their parents can affect their utilization of medical services. Finally, the patients who had visited medical institutions for musculoskeletal disorders were selected as the final subjects. The KHPS uses the additional diagnostic codes that were developed based on the Disease Classification System of Western and traditional Korean medicine. The diagnostic codes for musculoskeletal disorders were used to select the subjects. Following the International Classification of Diseases 10^th^ edition, the KHPS codes for musculoskeletal disorders are M00–M25 (arthropathies), M40–M54 (dorsopathies), M60–M79 (soft tissue disorders), M80–M94 (osteopathies and chondropathies), and M95–M99 (other disorders of the musculoskeletal system and connective tissue). There were a total of 3,630 subjects who had used outpatient services within 12 months of the survey.

### 3. Variables

We used Andersen’s model, a conceptual model illustrating the factors that lead to the use of health services, to select research variables to identify the determinants of traditional Korean medicine use by musculoskeletal patients. The model includes predisposing factors, enabling factors, medical need factors, and health behavior factors. We selected the survey items from the KHPS that are related to Andersen’s model.

The predisposing factors include sex, age, living with or without a spouse, and education level. Age was classified as under 40, 40–49, 50–59, 60–69, and over 70 years of age. Those living without a spouse included unmarried, separated, widowed, and divorced subjects. Education level was classified as less than elementary school graduate, middle school graduate, high school graduate, and some college or higher. Enabling factors included area of residence (i.e., living in a metropolitan city or not), type of health insurance, supplementary private health insurance, and average annual household income. The metropolitan cities included Seoul, Incheon, Busan, Gwangju, Daegu, and Ulsan. The type of health insurance was classified as National Health Insurance (NHI) and Medical Aid. Medical need factors included the presence of restrictions on daily life, the number of chronic diseases, disability status, hospitalization history, and the number of visits to conventional medical institutions. Patients were considered to have a history of hospitalization if they had received a hospital treatment within 12 months of the 2009 survey date. In order to classify the number of visits to conventional medical institutions, which is skewed data, we used the median value (4 times) to minimize the effect of extreme values. The number of visits to conventional medical institutions was classified as less than four and more than five. The number of times patients received outpatient services for conventional medicine did not include utilization of outpatient services for traditional Korean medicine. The health behavior factors included smoking, drinking, and regular exercise. Smoking behavior was divided into three categories: no history of smoking, past smokers, and current smokers. Drinking behavior was also divided into three categories: not drinking, drinking less than once a week, and drinking more than 2–3 times a week. Regular exercise was defined as participating in moderate or vigorous physical activities more than three days a week for at least 20 minutes at a time.

The dependent variables were the use of outpatient services and the frequency of traditional Korean medicine usage among musculoskeletal patients. The use of traditional Korean medicine was defined as a visit to a traditional Korean medical institution within 12 months of the survey date. The usage frequency was defined as the number of visits to traditional Korean medical institutions within the 12-month period.

### 4. Statistical Analysis

We conducted χ^2^ tests and t-tests to analyze the differences in traditional Korean medicine utilization based on the subjects’ characteristics. Because the utilization data was skewed and included patients who did not use traditional Korean medicine, the general medical utilization data was pre-processed using a two-part model [29], [30]. The first part of the two-part model involved logistic regression, estimating the probability of traditional Korean medicine use and excluding the musculoskeletal patients who had no experience using traditional Korean medicine. The second part, which includes multiple regression analysis, projects the frequency of traditional Korean medicine use. To reduce the skewness of the data, a natural logarithm was applied to the number of traditional Korean medical institution visits [31]. The level of statistical significance was set at 5%. All statistical analyses were conducted using SPSS 18.0 software (SPSS Inc., Chicago, Illinois, USA).

## Results

### 1. General Characteristics of the Subjects

Of the research subjects, 67.2% were women (Table 1). The smallest age group was the under-40 age group (8.1%), and the 60–69 age group was the largest at 28.9% (Table 1). Seventy-five percent of the subjects were living with their spouse, and 45.0% had less than an elementary school education (the most common education level). Residents of metropolitan areas comprised 39.4% of the total subjects, and most subjects (91.1%) had NHI (Table 1). Of the total number of subjects, 69.5% had supplementary private health insurance (Table 1). The average annual household income was approximately $24,755 (Table 1). The subjects with restrictions on daily life were 10.7% and subjects with disabilities were 10.3% (Table 1). The average number of chronic diseases was approximately 3.2 (Table 1). Fifteen percent of the subjects had been hospitalized for treatment within the past 12 months (Table 1). Fifty-five percent of the subjects visited conventional medical institutions for musculoskeletal disorders less than four times (Table 1). The majority of subjects (68.2%) had never smoked, 40.5% did not drink alcohol and 34.1% exercised on a regular basis (Table 1).

**Table 1:** General characteristics of the research subjects.

Variables	Subcategory	Total (%)	User of TKM (%)	Non-user of TKM (%)	χ^2^/t	*p*-value
Gender	Male	1,191 (32.8)	347 (29.6)	844 (34.4)	8.19	.005
	Female	2,439 (67.2)	826 (70.4)	1,613 (65.6)		
Age	<40	295 (8.1)	109 (9.3)	186 (7.6)	39.39	<.001
	40–49	518 (14.3)	207 (17.6)	311 (12.7)		
	50–59	796 (21.9)	289 (24.6)	507 (20.6)		
	60–69	1,049 (28.9)	302 (25.7)	747 (30.4)		
	≥70	972 (26.8)	266 (22.7)	706 (28.7)		
Living with spouse	Yes	2,723 (75.0)	879 (74.9)	1,844 (75.0)	.006	.967
	No	907 (25.0)	294 (25.1)	613 (24.9)		
Education level	Less than elementary school graduate	1,633 (45.0)	490 (41.8)	1,143 (46.5)	11.54	.009
	Middle school graduate	613 (16.9)	193 (16.5)	420 (17.1)		
	High school graduate	908 (25.0)	331 (28.2)	577 (23.5)		
	Some college or higher	476 (13.1)	159 (13.6)	317 (12.9)		
Residency in metropolitanarea	Yes	1,430 (39.4)	499 (42.5)	931 (37.9)	7.19	.008
	No	2,200 (60.6)	674 (57.5)	1,526 (62.1)		
Health insurance type	NHI	3,308 (91.1)	1,091 (93.0)	2,217 (90.2)	7.58	.006
	Medical Aid	322 (8.9)	82 (7.0)	240 (9.8)		
Supplementary PHI	Yes	2,522 (69.5)	881 (75.1)	1,641 (66.8)	25.90	<.001
	No	1,108 (30.5)	292 (24.9)	816 (33.2)		
Annual household income (mean ± SD) (US dollars)		24,755±22,609	26,574±22,775	23,887±22,484	−3.35	.001
Restriction(s) on daily life	Yes	389 (10.7)	102 (8.7)	287 (11.7)	7.90	.019
	No	3,240 (89.2)	1,071 (91.3)	2,169 (88.2)		
Number of chronic diseases (mean ± SD)		3.2±2.1	3.3±2.2	3.1±2.1	−2.21	.027
Disability	Yes	373 (10.3)	87 (7.4)	286 (11.6)	15.36	<.001
	No	3,257 (89.7)	1,086 (92.6)	2,171 (88.4)		
Hospitalization	Yes	543 (15.0)	154 (13.1)	389 (15.8)	4.56	.033
	No	3,087 (85.0)	1,019 (86.9)	2,068 (84.2)		
Number of visits to conventional medical institutions	≤4	1,960 (54.0)	710 (60.5)	1,250 (50.9)	29.79	<.001
	≥5	1,670 (46.0)	463 (39.5)	1,207 (49.1)		
Smoking	No smoking history	2,476 (68.2)	828 (70.6)	1,648 (67.1)	4.53	.102
	Past smoker	604 (16.6)	179 (15.3)	425 (17.3)		
	Current smoker	550 (15.1)	166 (14.2)	384 (15.6)		
Drinking	Not drink	1,469 (40.5)	451 (38.4)	1,018 (41.4)	6.50	.039
	Less than once a week	1,405 (38.7)	489 (41.7)	916 (37.3)		
	More than 2–3 times a week	756 (20.8)	233 (19.9)	523 (21.3)		
Regular exercise	Yes	1,239 (34.1)	428 (36.5)	811 (33.0)	4.28	.040
	No	2,391 (65.9)	745 (63.5)	1,646 (67.0)		
**Total**		**3,630 (100.0)**	**1,173 (32.3)**	**2,457 (67.7)**		

TKM, traditional Korean medicine; NHI, national health insurance; PHI, private health insurance; SD, standard deviation.

In analyzing traditional Korean medicine utilization, we found that the statistically significant predisposing factors were sex, age, and education level. A high percentage of the female users of traditional Korean medicine were younger than 60 years old and had a high education level (more than high school) (Table 1). The significant enabling factors were place of residency (metropolitan area or not), type of health insurance, supplementary private health insurance, and average annual household income (Table 1). Users of traditional Korean medicine were more likely to live in a metropolitan area, have NHI, have additional private health insurance, and have a higher annual household income than non-users. The medical need factors that yielded significant differences were restrictions on daily life, the average number of chronic diseases, disabilities, hospitalization history, and the number of visits to conventional medical institutions (Table 1). More people used traditional Korean medicine in cases where there were no restrictions on daily life, no disabilities, and no history of hospitalization. Traditional Korean medicine users had more chronic diseases and fewer visits to conventional hospitals (less than four times). Regarding health behavior factors, there were significant differences between participants related to drinking behavior and regular exercise (Table 1). The people who drank less than once a week and exercised regularly were more likely than other participants to use traditional Korean medicine.

### 2. Analysis of Traditional Korean Medicine Use by Musculoskeletal Patients Using a Two-part Model

The two-part model separately processes the use of traditional Korean medicine and the frequency of usage. We found three common influential factors: age, number of chronic diseases, and number of visits to Western medical institutions for musculoskeletal disorders. The probability of using traditional Korean medicine compared to Western medicine varied by age group: the 60–69 age group was 0.64 times (*p* = .007) less likely than the <40 age group (the standard) to use traditional Korean medicine, and the ≥70 age group was 0.64 times (*p* = .015) less likely than the <40 age group (Table 2). The musculoskeletal patients in the 40–49 age group and the ≥60 age group made significantly more visits to traditional Korean medical institutions than the <40 age group. Compared to <40 age group, the 40–49 age group, the 60–69 age group, and the ≥70 age group made 11.1% (*p* = .012), 19.2% (*p* = .001), and 27.4% (*p*<.001) more visits, respectively (Table 2). The number of chronic diseases was positively related to the use of traditional Korean medicine and the frequency of usage (14.6% higher) (Table 2). In the case of the number of visits to conventional medical institutions, the more visiting group (≥5) was 0.68 times (*p*<.001) less likely to use traditional Korean medicine than the less visiting group (≤4). However, the more visiting group also made 10.6% (*p*<.001) more visits to traditional Korean medicine institutions (Table 2). There was no relationship between the frequency of usage and residency in a metropolitan area, private health insurance coverage, and disability status; however, these three variables were positively related to the use of traditional Korean medicine overall (Table 2). Musculoskeletal patients with no disabilities who had private health insurance and lived in metropolitan areas preferred to use traditional Korean medicine rather than Western medicine (Table 2). The frequency of using traditional Korean outpatient services was 10.1% higher among the patients with restrictions on daily life and 8.5% lower in patients with a history of hospitalization (Table 2).

**Table 2:** Analysis of traditional Korean outpatient service utilization by musculoskeletal patients using a two-part model.

Variables	Subcategory	Logistic regression analysis			Multiple regression analysis	
		β	OR	*p*-value	β	*p*-value
Gender	Male					
	Female	.161	1.175	.074	.032	.316
Age	<40					
	40–49	.095	1.100	.544	.111	.012
	50–59	−.137	.872	.389	.073	.151
	60–69	−.450	.637	.007	.192	.001
	≥70	−.442	.643	.015	.274	<.001
Education level	Less than elementary school graduate					
	Middle school graduate	−.082	.921	.453	−.004	.902
	High school graduate	−.014	.986	.898	.065	.085
	Some college or higher	−.203	.816	.164	−.010	.786
Residence in metropolitan area	No					
	Yes	.163	1.177	.029	.048	.091
Health insurance type	Medical Aid					
	NHI	.227	1.255	.116	.031	.287
Supplementary PHI	No					
	Yes	.209	1.233	.031	.016	.633
Annual household income		.000	1.000	.657	.017	.597
Restriction(s) on daily life	No					
	Yes	−.162	.851	.216	.106	<.001
Number of chronic diseases		.121	1.129	<.001	.146	<.001
Disability	No					
	Yes	−.352	.703	.010	.004	.882
Hospitalization	No					
	Yes	−.169	.845	.117	−.080	.005
Number of visits to conventionalmedical institutions	≤4					
	≥5	−.383	.682	<.001	.106	<.001
Drinking	Not drinking					
	Less than once a week	.018	1.018	.838	.019	.546
	More than 2–3 times a week	−.076	.928	.496	.012	.723
Regular exercise	No					
	Yes	.103	1.108	.189	−.028	.326
		χ^2^ = 132.20, df = 20 (*p*<.001)			F-value = 7.92 (*p*<.001)	
		−2 Log likelihood = 4432.11			R^2^ (adj. R^2^) = .121 (.106)	
		Number of observations = 3,630			Number of observations = 1,173	

OR, odds ratio; NHI, national health insurance; PHI, private health insurance.

## Discussion

The proportion of musculoskeletal patients who used traditional Korean medical outpatient services was 32.3% based on an analysis of the KHPS data from 2008 and 2009. This utilization rate is considerably higher than the proportion of individuals who use traditional Korean medicine according to the Korean NHI claims database (approximately 5% [11]). Musculoskeletal disorders have direct causes, such as an increase in simple repetitive tasks, computer use, and improper working postures, and they also have indirect causes, such as lifestyle and exercise [32]. In particular, the excessive use of muscles in exercise and repetitive tasks can cause tendon and ligament weakness and chronic pain, and the younger subjects who participated in many social activities tended to have these problems [32]. According to the medical benefits of traditional Korean medicine from 2007 to 2009, NHI claims 68.0% were related to musculoskeletal disorders [11]. Also, previous studies reported that the utilization rate of traditional Korean medical services among patients with arthritis was higher than the rate among patients with other chronic diseases [33] and that the most frequent users of traditional Korean medicine were musculoskeletal patients [18], [34], [35]. In the U.S. and the U.K., the most frequent CAM users were musculoskeletal patients who suffered from back pain or osteoarthritis [17], [36]. In Japan, the major cause for using CAM was musculoskeletal problems [37]. In addition, the utilization rate found in this study was higher than the rate found in other researches about traditional Korean medicine utilization for musculoskeletal disorders [18], [19]. This difference may be related to the different lengths of the medical utilization periods. In previous researches, researchers set the utilization period at 2 weeks or 6 months, but we set it at 12 months. The 12-month period is longer than the periods used in previous studies, which is why the utilization rate in our study seems high.

In this research, we used a two-part model to identify the determinants of the use of traditional Korean outpatient services among musculoskeletal patients. The number of visits to traditional Korean medical institutions increased among elderly (≥60) patients with restrictions on daily life, more chronic diseases, no history of hospitalization, and more visits to conventional hospitals (≥5). The frequency of traditional Korean medicine usage in the ≥60 age group and in the 40–49 age group were roughly 20% and 11.3% higher than the <40 age group, respectively. Although it was not a statistically significant difference, the frequency of usage for patients aged 50–59 years was 7.4% higher. It has been assumed that the positive correlation between age and the frequency of usage is rooted in the positive sentiment that elderly Koreans have towards traditional Korean medicine [38], [39]. Previous researches found a greater preference for CAM among young females [40]–[42]. However, these researches only considered variables related to the validity of CAM use and neglected variables related to the level of utilization (i.e., frequency of usage and cost). Our study result shows that the frequency of traditional Korean medicine use was even higher in the older group than in the younger group. There also seem to be other influencing factors, namely that seniors generally use more health care than young people and that the NHI system reduces the deductible (or the out-of-pocket costs) for seniors over 65 years old [43].

Musculoskeletal patients with restrictions on daily life made more visits to traditional Korean medical institutions. Previous research has already shown the elderly with limited mobility use more outpatient services [44]. It should be noted, however, that the relationship between the restriction variable and the frequency of traditional Korean medicine usage should be considered with skepticism. Restriction was loosely defined, unlike the variables that were defined by a clinical standard (e.g., disability and hospitalization). Thus, the accuracy of the variable is questionable.

The number of chronic diseases was also a common determinant of the use of traditional Korean medicine and the frequency of usage. The probability of use and the frequency of traditional Korean medicine usage increased as the number of chronic diseases increased. Ongoing management and conservation treatments are required for chronic disease care because chronic diseases are difficult to cure completely with conventional Western medicine. Patients are dissatisfied with conventional treatments when these treatments are ineffective, produce side effects, are viewed as too technologically oriented (or impersonal), or are too expensive [45]. Other studies have also documented that people use CAM because of their dissatisfaction with conventional medicine [12], [17], [36], [46]. In addition, chronically ill patients may prefer to explore medical institutions that provide high quality services and demonstrate an interest in health [47]. Previous researches have also found that CAM utilization is high among chronically ill patients [2], [3], [48], [49].

Musculoskeletal patients with no history of hospitalization made more visits to traditional Korean medical institutions. Previous researches show that patients who require ongoing management but not hospitalization, such as patients with depression, obesity, and asthma, prefer to use CAM [13], [45], [50]. Lim et al. (2005) found that people only prefer CAM over conventional medicine for non life-threatening conditions, such as minor musculoskeletal disorders and digestive tract illnesses [51]. Also, Yamashita et al. (2002) found high usage of CAM among patients with low-severity conditions [52]. This study result is consistent with the previous CAM research; the frequency of traditional Korean medicine usage is high among musculoskeletal patients with low-severity conditions and no history of hospitalization.

Healthcare system factors (i.e., health insurance type and supplementary private insurance) did not have a statistically significant effect on the use of traditional Korean medicine. Korean NHI coverage for traditional Korean medicine is very limited [53]. Patients with supplementary private health insurance would pay less out-of-pocket costs. We expected to see a higher frequency of using traditional Korean medicine among the musculoskeletal patients with supplementary private health insurance, but this result was not found. It seems that most traditional Korean medical services for musculoskeletal patients are low-cost treatments, such as acupuncture, moxibustion, and physical therapy, and these services may not impose a large financial burden on the patients.

Musculoskeletal patients who paid more frequent visits to (≥5) conventional hospitals or clinics also more frequently used traditional Korean medicine than the less visiting group (≤4). If people who use routinely conventional medicine start to use traditional Korean medical services, the total utilization of outpatient services increases because traditional Korean and conventional medicine are both used. This finding suggests the possibility that musculoskeletal patients use traditional Korean medicine as a complement to Western medicine rather than an alternative form of treatment. However, there could be problems caused by using Western and traditional Korean medical services together. For example, patients may lose time and money receiving duplicate or different prescriptions for the same disease and symptoms, or they may not receive proper care in a timely manner [54]. To prevent these problems and allow traditional Korean medicine to perform a substantial complementary role, integrated care and cooperation between Western and traditional Korean health care providers are required. In Korea, medical laws were recently revised to allow cooperative treatment between Western and traditional Korean medicine. In addition, the number of medical institutions that claim to support cooperative treatment systems is increasing. However, it is difficult to find an example of a substantive cooperative care system in which Western and traditional Korean medical providers discuss treatment plans together [55].

In this study, we analyzed secondary data from the KHPS to meet the research objectives. We could not, however, include some variables that might affect traditional Korean medicine utilization, such as patients’ evaluation of medical services, patients’ satisfaction with medical services, and factors related to medical providers. This limitation seems to be one of the reasons why the explanatory power of our model was low for the frequency of traditional Korean medicine usage. In previous studies, personal characteristics of users and belief in or satisfaction with CAM were found to influence CAM use or usage frequency [13], [17], [45]. These variables should be included in future research that analyzes CAM utilization. Variables related to medical providers also affect the frequency of traditional Korean medicine usage. Korea has adopted fee-for-service as a payment model for the NHI. If health care providers increase the types and amount of provided medical services to their patients, they receive more money from the NHIC. Providers can induce patients to return for visits or they can provide more services than necessary to increase their revenue. Therefore, it is necessary to consider factors related to health care providers in future research. Finally, the KHPS asked respondents about their use of health services for the 12 months prior to the survey date, so there could be recall bias.

### Conclusions

This research analyzed the individual characteristics that affect traditional Korean medicine use by musculoskeletal patients. With the results of this research, we confirmed that many musculoskeletal patients have used traditional Korean medicine, and the important determinants of the frequency of traditional Korean medicine usage are age, restrictions on daily life, the number of chronic diseases, hospitalization history, and the number of visits to conventional hospitals and clinics for musculoskeletal disorders. The results help clarify the characteristics of traditional Korean medicine users and may be used as a basic resource for related policymaking by government officials and healthcare professionals.
